# Atlantic salmon (*Salmo salar*) under challenge: Heart rate and acceleration dynamics during exercise and stress

**DOI:** 10.3389/fphys.2025.1562665

**Published:** 2025-04-07

**Authors:** Wisdom E. K. Agbeti, Arjan P. Palstra, Suzy Black, Leonardo Magnoni, Martin Lankheet, Hans Komen

**Affiliations:** ^1^ Animal Breeding and Genomics, Wageningen University and Research, Wageningen, Netherlands; ^2^ Plant and Food Research, Nelson, New Zealand; ^3^ Experimental Zoology Group, Wageningen University and Research, Wageningen, Netherlands

**Keywords:** swimming physiology, oxygen consumption, sensor implants, fish behavior, aquaculture

## Abstract

This study investigated the heart rate (HR) and acceleration (AC) dynamics of Atlantic salmon (*Salmo salar*) during a swim fitness test in a swim tunnel. Experimental fish were implanted with data loggers equipped with HR and AC sensors. These fish, and controls that were not implanted, were subjected to a swim-fitness test at incremental speeds from 0.2 to 1.0 m.s^–1^. Oxygen consumption (MO_2_) and locomotory behavior were monitored. Subsequently, these fish were subjected to a stress challenge test to further study the link between induced stress and HR and AC. When swimming from 0.2 to 1.0 m.s^–1^, the HR of implanted fish (N = 19) was high in the swim tunnels but remained stable between 82 and 84 beats per minute (bpm), despite significant increases in MO_2_, AC, tail beat frequency (TBF), and head width frequency (HWF). The stable HR observed was also reflected by HR explaining only ∼15% of the variation in MO_2_. MO_2_ of implanted fish increased from 238 to 343 mg.kg^–1^.h^–1^ when swimming from 0.4 to 1.0 m.s^–1^. With increasing swimming speeds, AC values of implanted fish increased from 16 to 27 milli-g and explained ∼40% of the variation in MO_2_. TBF increased linearly with swimming speed, and from 0.4 m.s^–1^ onward, it correlated strongly with MO_2_, similarly for HWF. Under controlled stress conditions, the HR values of fish were significantly higher than baseline conditions but similar during stress regardless of intensity. Also, AC showed similar stress peak patterns as HR. From these results, we conclude that the increased oxygen demand when swimming at increasing speeds is not met by increasing HR alone in Atlantic salmon. This supports the hypothesis that stroke volume plays an important role in boosting cardiac output. AC, in contrast to HR, showed a strong positive correlation with MO_2_ during swim-fitness tests and may serve as a reliable predictor of energy expenditure. TBF and HWF may also be useful predictors, but HR is not in Atlantic salmon. HR did show positive responses to induced stress but, similar to swimming, up to maximum values under 90 bpm.

## 1 Introduction

Fish spend energy when swimming, and this expenditure is believed to be a significant component of their overall energy budget (Webb, 1975; Magnoni et al., 2013). Fueling swimming activity is primarily supported by aerobic metabolic pathways, with adenosine triphosphate as the energy source (Webb, 1975; Videler, 1993; Videler and Weihs, 1982). Maximum metabolic capacity can be linked to physiological fitness and is typically quantified indirectly by measuring oxygen consumption (MO_2_) under controlled laboratory conditions using swim tunnels (Videler, 1993; Arechavala-Lopez et al., 2021; Beamish, 1978; Brett, 1964). Swim tunnels equipped with oxygen electrodes and high-speed cameras can be used to assess swimming performance and estimate the contributions of aerobic metabolism to overall energy expenditure. Swim fitness tests involve gradually increasing the swimming speed of fish [e.g., (Palstra et al., 2008)]. As swimming speed increases, acceleration (AC) and locomotory parameters such as tail beat frequency (TBF) and tail beat amplitude (TBA) also increase (Palstra et al., 2024; Hvas et al., 2021a; Agbeti et al., 2024). Swimming faster increases the demand for oxygen by the slow (or red) skeletal muscle which is necessary to produce adenosine triphosphate until the gait transition point is reached where fish may use anaerobic mechanisms to meet this energy demands. The increase in oxygen demand would require increased cardiac output (Videler, 1993; Altringham and Ellerby, 1999; Eliason et al., 2013). Heart rate (HR), as a key cardiac feature, serves as an important physiological indicator of a fish’s capacity to transport oxygen to tissues (Farrell et al., 2009) and adaptation to environmental and physiological challenges (Svendsen et al., 2021). By regulating HR, along with other cardiac variables such as stroke volume, fish can modulate their cardiac output (Farrell et al., 2009; Thorarensen et al., 1996), thereby optimizing oxygen delivery to meet the energetic requirements of activity. HR can be monitored under controlled conditions to infer cardio-respiratory performance of fish (Palstra et al., 2024; Hvas et al., 2021a; Yousaf et al., 2024).

The advent of bio-logging sensors has revolutionized the study of fish physiology (Bjarnason et al., 2019). The use of such bio-loggers ensures the continuous monitoring of metrics like HR and AC in freely swimming fish for prolonged durations (Palstra et al., 2024; Svendsen et al., 2021; Clark et al., 2010; Zrini and Gamperl, 2021). Therewith, bio-loggers can be used to enhance our understanding of the impacts of climate change on aquatic ecosystems (Eguiraun and Martinez, 2023; Føre et al., 2018). By facilitating data collection in natural settings, these sensors provide insights into active metabolic rate, behavior and stress responses of fish. However, species-specific calibrations are necessary to ensure accurate assessment of these parameters. This involves conducting laboratory studies to link HR and AC to swimming speed and MO_2_ to gain deeper understanding on how these relations change as activity levels increase. In an earlier study, carried out under steady and unsteady flow conditions, we used Atlantic salmon (*Salmo salar*), implanted with acoustic transmitter tags, as a model to investigate oxygen consumption and AC dynamics of fish (∼250–315 g) in relation to swimming speed increments (Agbeti et al., 2024). However, we did not quantify HR dynamics during increased swimming activity under these flow conditions. In this follow-up study, we used data loggers to investigate HR and AC during a swim-fitness test under steady flow conditions.

Atlantic salmon are known for their ability to migrate long distances and they are one of the most highly valued aquaculture finfish species (Asche and Bjorndal, 2011). Currently, grow-out production sites for this species and other farmed marine fish species are primarily located in sheltered near-shore areas. These areas are vulnerable to extreme weather events caused by climate change (Griggs and Reguero, 2021). Consequently, there is a growing interest in moving aquaculture operations to more exposed marine environments which are less prone to these events (Morro et al., 2021; Holmer, 2010) but characterized by strong waves and currents. Further knowledge is needed on the physiological performance of fish under culture conditions taking into account fish welfare considerations to be applied in precision farming.

For this study, DST milli-HRT ACT Starr-Oddi loggers were used to record HR and AC of fish during a swim-fitness test. Additionally, high-speed cameras were employed to monitor locomotory behavior, and electrodes were used to measure MO_2_. The main objective of the study was to investigate the HR and AC dynamics of post-smolt Atlantic salmon during incrementally increased swimming exercise in a swim tunnel. We hypothesize that with increasing swimming speed, HR and AC of post-smolt Atlantic salmon will increase in positive relation with MO_2_, as the HR should ensure sufficient oxygen delivery to the skeletal muscles. Subsequently, these fish were subjected to a stress challenge test involving four sequential stressors of increasing intensity to further study the link between induced stress and HR and AC. This challenge test has been described by Svendsen et al. (2021) for Atlantic salmon and applied by Palstra et al. (2024) for yellowtail kingfish (*Seriola lalandi*). We hypothesize that HR and AC of post-smolt Atlantic salmon will increase with induced stress intensity, reflecting the fish’s physiological adaptations to manage accumulative stress loads.

## 2 Materials and methods

### 2.1 Experimental fish and conditions

Atlantic salmon smolts (N = 36, ∼300 g) were provided by Aquafuture (Hagen, Germany) and transported to the Wageningen University and Research animal experimental facilities (CARUS, Wageningen, Netherlands). Upon arrival, fish were allowed to acclimatize for at least 2 weeks, in three circular holding tanks of a volume 1,000 L with shelter area, supplied with well-aerated brackish water (15 ppt) at 14°C ± 1°C, and connected in a recirculating aquaculture system. Briefly, the setup included a sump, settling tank, drum filter, protein skimmer, and trickling filter for efficient operation. Within the 2 weeks of acclimation, salinity was gradually increased by 1.35 ppt per day from 15 to 34 ppt. During acclimation, fish were kept at a 12 h:12 h light:dark regime and hand-fed with a commercial pellet (crude protein 43%, crude lipid 29%, ash 7%, 5 mm). After the acclimation period, feeding was done using automatic belt feeders and water temperature was gradually reduced from 14°C ± 1°C to 12°C ± 1°C with 0.5°C per day. After 2 months, fish (N = 20, ∼471 g) were large enough to be randomly selected for surgical implantation of the loggers. The remaining N = 16 fish (∼410 g) were considered as the controls without an implanted logger. Groups were kept separate over the three tanks. Water quality over the whole experimental period remained well in the tanks with values of NH_4_^+^ 0.15 ± 0.02 mg.L^–1^, NO_2_^−^ 0.3 ± 0.1 mg.L^–1^, and NO_3_^−^ 35 ± 4 mg.L^–1^.

### 2.2 Heart rate and acceleration loggers

The DST milli-HRT ACT loggers from Starr-Oddi (Gardabaer, Iceland) were surgically implanted in the fish of mean ± SE body weight (BW) = 470.8 ± 11.1 g; standard length (SL) = 31.8 ± 0.3 cm, and total length (TL) = 35.5 ± 0.4 cm. Each logger measured 3.9 cm in length, 1.3 cm in width, and weighed 12 g, was airtight and biocompatible with fish. Fish were anesthetized with 0.3 mL.L^–1^ of phenoxyethanol in aerated seawater, and then placed ventral side up on a surgical table under a continuous low-dose phenoxyethanol flow (0.15 mL.L^–1^) over the gills. Lidocaine (100 μL; 2 mg.kg^–1^) was injected locally for pain relief before a 1–2 cm incision was made on the ventral side near the pelvic fins. After inserting the disinfected logger into the abdominal cavity towards the pericardial cavity, it was anchored with a two-knotted single suture (Ethilon 3-0 669H; FS-1 24 mm 3/8c reverse cutting needle and 75 cm black monofilament non-absorbable suture). The incision was stitched with two single sutures and treated with betadine. Fish were anesthetized for approximately 2 ± 1 min, surgery lasted 7 ± 2 min, and fish resumed swimming after 2 ± 1 min. Implanted fish recovered for 10 days in a separate tank before experimentation.

Post-experiment, implanted fish were euthanized with an over-dose of phenoxyethanol and loggers were retrieved. HR was derived from electrocardiogram (ECG) signals recorded at 200 Hz, every 10 min for 7.5 s, expressed as beats per minute (bpm). The quality of the ECG signal was evaluated using the quality index (QI) provided by the on-board logger algorithm. A QI value of 0 (QI_0_) denotes very good signal quality, while QI_1_ and QI_2_ indicate decreasing lower quality. A QI_3_ value means no R–R interval was detected, and HR values associated with QI_3_ were excluded from the analysis. For ECG signals with QI_1_ or QI_2_ values, the HR data were further reviewed, and manual calculations were performed when needed. Six HR measurements were recorded for each swimming speed. Baseline HR and AC were the average of 30 recordings made hourly before the first swimming trial. AC was measured as average external AC value (in milli-g-force) recorded at 10 Hz, and calculated as the vectorial sum of dynamic body acceleration by subtracting static acceleration (due to gravity) from raw AC data, averaged over 1 min (600 measurements).

### 2.3 Swim-fitness test and respirometry

The swimming experiment was conducted using Blazka-type swim-tunnels, described in detail by Van Den Thillart et al. (2004). These tunnels were constructed from Perspex with dimensions of approximately 28.8 cm in diameter, 200 cm in length, and with a volume of 127 L. The tunnels were regulated by a Siemens Micro Master (Basic 370) digital power and frequency controller. To maintain high oxygen levels, the tunnels were linked to a 400-L tank filled with aerated system water. An EHEIM pump with a capacity of 600 L/H (Universal; EHEIM GmbH & Co. KG, Deizisau, Germany) circulated water from the tanks through the tunnels. A valve could close the water inlet to measure oxygen content decline. A bypass with an oxygen probe in a 4-channel respirometry system (DAQ-PAC-G4; Loligo Systems Aps, Tjele, Denmark) enabled the measurement of total oxygen content and subsequently the decline in oxygen content as consumed by the fish (ΔO_2_%).

Fish, referred to as ‘implanted fish’ when a logger was surgically implanted and “control fish” when not, were swum in series of four in random order and active metabolic rate was measured. Each fish was individually removed from the holding tank, slightly anesthetized, and was measured for TL, SL and BW before being introduced into the swim-tunnels. After a recovery and acclimation period of 1 h, a critical swimming speed (U_crit_) protocol was executed starting without propeller activity, and then with propellor activity inducing swimming speeds from 0.2 up to 1.0 m.s^–1^. Swimming speeds were increased with increments of 0.2 m.s^–1^, and fish were swimming 60 min at each speed. Before swimming speed was increased, tunnels were flushed for 10 min to re-establish high oxygen levels. Fish were allowed to acclimatize to a newly set swimming speed for 10 min before oxygen measurements commenced. Hence, oxygen measurements were done for 40 min per swimming speed. The investigator was continuously present to observe swimming behavior which was also monitored with high-speed cameras. The swim trial was terminated when a fish fatigued, determined as the point when fish touched the rear grid of the swim tunnel for more than 20 s and could not be stimulated to swim within this period. The fish was then removed from the tunnel and transferred to a recovery tank. The exact time of fatigue was recorded and used to calculate the critical swimming speed (U_crit_) according to Brett (Brett, 1964) and Plaut (Plaut, 2001) as follows:

$$U_{crit}=U_{i}+\left[U_{ii}*\left(T_{i}/T_{ii}\right)\right]$$

where U_crit_ is the critical swimming speed in m.s^–1^, (absolute U_crit_), U_i_ is the highest velocity completed before exhaustion in m.s^−1^, U_ii_ is the prescribed velocity increment in m.s^−1^, T_i_ is time to fatigue at final velocity level in minutes, and T_ii_ is the prescribed time interval (= 60 min)

Background oxygen consumption (tunnels without fish) was measured at all swimming speeds and extracted from the values measured with fish present. The solid blocking effect was calculated (Bell and Terhune, 1970) but was negligible at this fish size in these tunnels. From the decreasing oxygen contents in the tunnels, the oxygen consumption (MO_2_ in mg.kg^–1^.h^–1^) and cost of transport (COT; in mg. kg^−1^. km^−1^.) were calculated using the following formulas:

$$MO_{2}=(\frac{∆O_{2}\left(DO\max\;*V/100\;\right)}{BW*t}$$

where $∆O_{2}$ is percentage oxygen saturation; DOmax (mg.L^–1^, accounting for temperature and salinity) is the maximum amount of oxygen dissolved in the seawater; V is the volume of swim tunnel (127 L); BW (kg) is body weight of the fish; and *t* is the time in hours.

$$\mathrm{COT}=\frac{MO_{2}}{∆d\;}\;1000$$

where MO_2_ is the consumed oxygen (mg. kg^−1^. km^−1^.), and ∆d is the distance covered in meters (m) as calculated from the flow speed and exposure time.

In these calculations, the logger weight of 12 g was not subtracted from the BW of the implanted fish.

Optimum swimming speed was determined by plotting a two-degree polynomial trend line through COT values vs. swimming speeds. The point on this trend line with the lowest COT was calculated by equaling the first derivative to zero (Palstra et al., 2015).

The first N = 10 implanted fish were immediately dissected to secure the logger data after the swim test. These fish were euthanized in 0.7 mg.L^−1^ of phenoxyethanol in system seawater, dissected, the loggers extracted and the data downloaded. Implanted fish (N = 7), after 8 days of recovery from swim test, were subjected to the stress challenge test described in Section 2.5.

### 2.4 Locomotory behavior

A Basler 2040-90um NIR USB3 camera was used to collect the high-speed video footage during salmon locomotion in the swim tunnel. The camera was mounted 1 m below the center of the swim tunnel and the camera’s visual field was adjusted to cover the entire swim section of the swim tunnel. Video recording at each swimming speed was done at a frame rate of 25 frames per second with a 15 ms exposure time. Video analysis was done by first binning pixels 2 × 2 to improve its sensitivity by a factor of 4. The resolution of the final images was 14.25 pixels per cm, and their overall dimensions were 1024 × 512 pixels. Fish contours were detected in real-time using Python-based software that incorporated the OpenCV image analysis library [see Figure 2 in Arechavala-Lopez et al. (2021)]. A series of image processing methods were employed to detect fish, starting with a 3-pixel median filter and a 5-pixel Gaussian blur to minimize noise, then histogram normalization to boost contrast, and finally a luminance threshold to differentiate dark fish from the light background.

Following the detection of objects using the “find contours” algorithm, fish selection was based on their surface area and the length-to-width ratio of an ellipse fitted to the contour. A Kalman filter in OpenCV provided smoothed estimates of the fish’s trajectory, determined by the contour’s center of mass, along with timing data. Complete body contours were saved for subsequent analysis. The midline of fish was analyzed by using a distance transform, quantifying the nearest distance to the contour for each pixel. The tip of the snout was found by fitting a line to midline points in the fish’s anterior region [refer to Figure 2 in Arechavala-Lopez et al. (2021)]. The snout was detected as the first point outside the contour along the fitted midline. The ridge of the distance transform’s maxima was traced from the snout in 0.7 cm steps to establish the complete axis of the fish. The maxima were tracked by continually identifying the highest point within a 0.7 cm radius circle centered on the previous point and clearing the circle’s values to avoid any reversal in direction. This was done until the tail tip was reached. Afterwards, the axis obtained was slightly smoothed using a univariate spline, with a spline order of 3 and a smoothing factor of 5, to reduce the impact of any contour irregularities on the midline.

Tailbeat parameters were obtained by selecting a point in the tail that was 14.0 cm away from the snout and measuring its lateral excursion relative to the midline through the head. We obtained tailbeat frequency and amplitude by analyzing the tail excursion as a function of time through spectral analysis. Spectrograms were created by calculating temporal windows with a size of 1.28 s (32 frames), which were then shifted frame by frame. To increase frequency domain resolution, the signal was padded with zero values to a width eight times that of the original signal. Frequency and amplitude were determined at each frame by identifying the maximum value in the spectrogram. A similar approach was used to calculate head width frequency (HWF) and amplitude (HWA). HWF represents the frequency of width oscillation of the head region at the location of the opercula and serves as a proxy for the rhythmic movements of the opercula, indicative of respiration rate. HWA measures the amplitude of head-width modulations.

### 2.5 Stress challenge test and cortisol

During the stress challenge, a group of N = 7 implanted fish was placed in a 1000 L tank together with control fish and subjected to four different stressors with ascending level of intensity. These included: (i) reducing water level and immediately filling it up, (ii) reducing water level and filling it up after 1 minute, (iii) reducing water level and filling it up after 5 minutes, and (iv) reducing water level and filling it up after 5 minutes while also chasing the fish with a net, following the protocol published by Svendsen et al. (2021). Each stress induction was initiated 60-min after the previous one. It took approximately 4 min to drain the tank until the dorsal fins of the fish were exposed, and approximately 11 min to refill the water to its maximum level. Consequently, the time intervals between the four stress inductions were 45 min, 44 min, and 40 min, respectively. The loggers were programmed to record HR and AC every 10 min. As such, the first 20 min (corresponding to two data points) were assigned to the stress induction period, while the subsequent 40 min (corresponding to four data points) represented the intermittent recovery period before the next stress induction. Just before increasing the water level in each step, a 1 L water sample was taken and stored in a 1 L glass bottle at −20°C for later cortisol measurements as described by Palstra et al. (2024). Briefly, cortisol was concentrated using Oasis HLB extraction cartridge (186000132; Waters, Milford, CT, United States), pre-conditioned with 5 mL methanol, raised with 5 mL MilliQ water and eluted with 3 mL ethanol. After evaporating the ethanol, the residue was reconstituted in 100 μL water, followed by liquid-liquid extraction with 2 mL diethyl ether. This extraction was repeated four times, and the final dried (steam air at 45°C) residue was dissolved in 200 μL assay buffer for cortisol ELISA. Extracts were 1:4 times diluted and analyzed as duplicates by Fish Cortisol ELISA Kit (CSB-E08487F_96; Cusabio, Houston, TX, United States). HR and AC were recorded a day before the stress test from 10:00 to 17:00, serving as baseline values. The stress challenge test was conducted within the same time frame, and HR and AC were recorded during this period. After stress test, HR and AC were also recorded for an additional 3 h.

### 2.6 Statistics

Data analysis was conducted using R statistical software (v 4.3.0, https://www.r-project.org/). The lme4 (Bates et al., 2015) and lmerTest (Kuznetsova et al., 2017) packages were utilized for model fitting. Normality and homoscedasticity of the data were assessed. A Student’s t-test was performed to identify potential differences in biometric parameters (BW, SL, and TL), U_opt_, COT_min_, and U_crit_ between implanted fish and control groups. A linear mixed model (LMM), fitted using restricted maximum likelihood (REML), was applied to examine the relationships between MO_2_, HR, AC, and locomotory parameters with swimming speed, as well as to assess the impact of logger implantation. The general model was:

y=Xβ+Zb+e

Where y denotes the vector for one response variable (MO_2_, HR, AC, TBA, TBF, HWA, and HWF); X is the design matrix associated with the explanatory coefficients β, which account for swimming speed and the presence of a logger. Z corresponds to the design matrix of the random effects coefficients b, representing individual fish, and e stands for the vector of random errors. The model assumes that the explanatory variables and random errors are mutually independent, normally distributed and identical. We also tested for interaction effect of the explanatory variables and included it in the model when found significant. Further, LMM was also employed to evaluate whether HR, AC, and locomotory parameters were effective predictors of MO_2_. HR and AC in relation to the four consecutive stressors were also tested using LMM. Biometric parameters were included as covariates in all models. Explanatory variables that were not significant were removed from the models. Statistical significance was set at p < 0.05, and all values are presented as means ± SE.

## 3 Results

### 3.1 Biometric parameters

The groups of implanted fish had a mean TL of 35.48 ± 0.37 cm and the control fish had a mean TL of 34.63 ± 0.28 cm. TL did not differ significantly between the two groups (t-test, p > 0.05, df = 33.24, t-value = 1.9). Similarly, the mean SL of 31.75 ± 0.33 cm for implanted fish was not significantly different from that of control fish of 30.91 ± 0.24 cm (t-test, p > 0.05, df = 32.8, t-value = 2.12). However, although randomly selected, a substantial difference in BW existed (t-test, p < 0.05, df = 33.64, t-value = 4.32) with the implanted fish (471 ± 11 g) being slightly heavier than control fish (411 ± 9 g).

### 3.2 Respirometry

**FIGURE 1:**
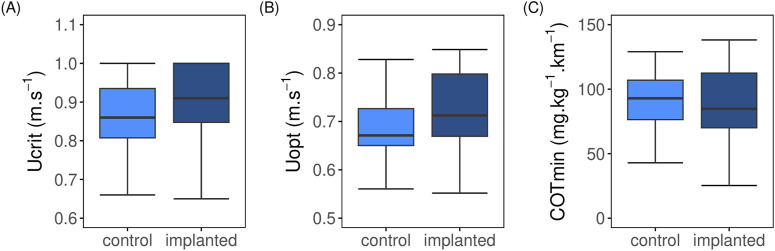
Comparison of swimming performance parameters between control (light blue; N = 16) and implanted (dark blue; N = 20) post-smolt Atlantic salmon (*Salmo salar*) swum from 0.2 to 1.0 m.s^–1^ under steady flow condition in swim tunnel. **(A)** Absolute critical swimming speed (U_crit_; m.s^–1^); **(B)** Absolute optimum swimming speed (U_opt_; m.s^–1^); **(C)** Minimum cost of transport (COT_min_; mg.kg^–1^.km^–1^). No significant difference exist between implanted and control fish (t-test, p > 0.05).

At the highest swimming speed tested, 12 implanted fish and 13 control fish fatigued. Implanted fish exhibited a U_crit_ ranging from 0.65 to 1.0 m.s^–1^, with a mean U_crit_ of 0.84 ± 0.03 m.s^–1^ (Figure 1A), equivalent to 2.62 ± 0.09 BL.s^–1^ for fish that fatigued. This mean U_crit_ was statistically comparable (t = 1.04, df = 29.31, p = 0.31) to that of the control fish (0.83 ± 0.03 m.s^–1^, corresponding to 2.66 ± 0.08 BL.s^–1^), which ranged from 0.66 to 1.0 m.s^–1^. No significant differences was found in mean U_opt_ (t = 0.26, df = 29.45, p > 0.05; Figure 1B) of implanted (0.72 ± 0.02 m.s^–1^) and control (0.71 ± 0.02 m.s^–1^). Similarly, mean COT_min_ values (t = 0.47, df = 33.96, p > 0.05; Figure 1C) were comparable between the two groups.

**FIGURE 2:**
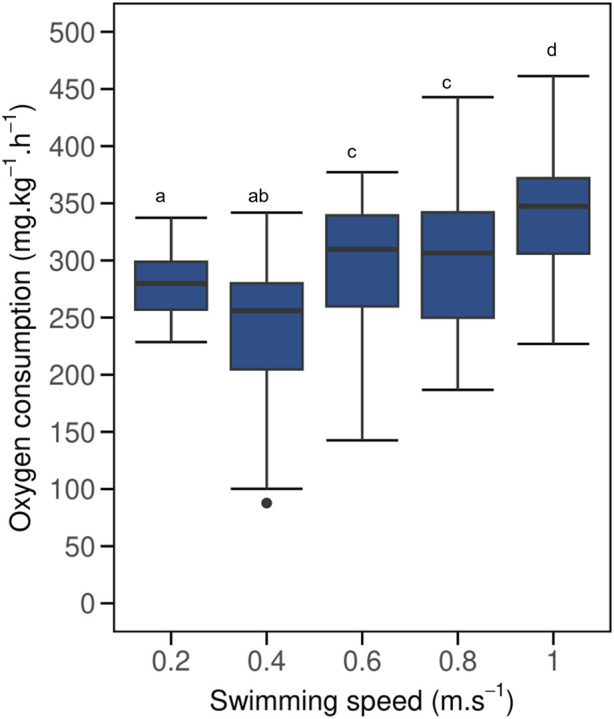
Boxplot of oxygen consumption (MO_2_) for implanted post-smolt Atlantic salmon (*Salmo salar*) swimming at increasing swimming speed under steady flow condition in a swim tunnel. Each boxplot represents N = 20 fish. The black dot in the boxplot represent outliers. Mean MO_2_ increased when swimming at speeds of 0.4–0.6 m.s^–1^, but remained similar when swimming at speeds of 0.6 and 0.8 m.s^–1^, before significantly increasing again at a swimming speed of 1.0 m.s^–1^ (LMM p < 0.05). Different lowercase letters indicate significant difference between values (LMM p < 0.05); same letters denote otherwise. A similar graph for control fish is included in Supplementary Figure S1.

MO_2_ of implanted fish were not affected by logger implantation and BW differences when compared to control fish (Figure 2; Supplementary Figure S1, LMM p > 0.05, AIC = 1641; Supplementary Table S1). Mean MO_2_ of implanted fish (Figure 2) increased with increasing swimming speed from 238 ± 17 to 343 ± 19 mg.kg^–1^.h^–1^ when swimming from 0.4 to 1.0 m.s^–1^ respectively (R^2^ = 0.91; y = 159.72x + 182.72). Mean MO_2_ of implanted fish decreased slightly when swimming at 0.4 m.s^–1^ compared to when swimming at 0.2 m.s^–1^, though the difference was not significant (LMM p > 0.05). Swimming speed of 0.6–1.0 m.s^–1^ had significant effect on MO_2_ (LMM p < 0.05) in comparison to 0.4 m.s^–1^. However, MO_2_ of implanted fish at 0.6 (297 ± 15 mg.kg^–1^.h^–1^) and 0.8 m.s^–1^ (301 ± 17 mg.kg^–1^.h^–1^) remained statistically similar (LMM p > 0.05, AIC = 708, Supplementary Table S1). This was followed by a substantial increase at 1.0 m.s^–1^ (LMM p < 0.05).

### 3.3 Swimming behavior

**FIGURE 3:**
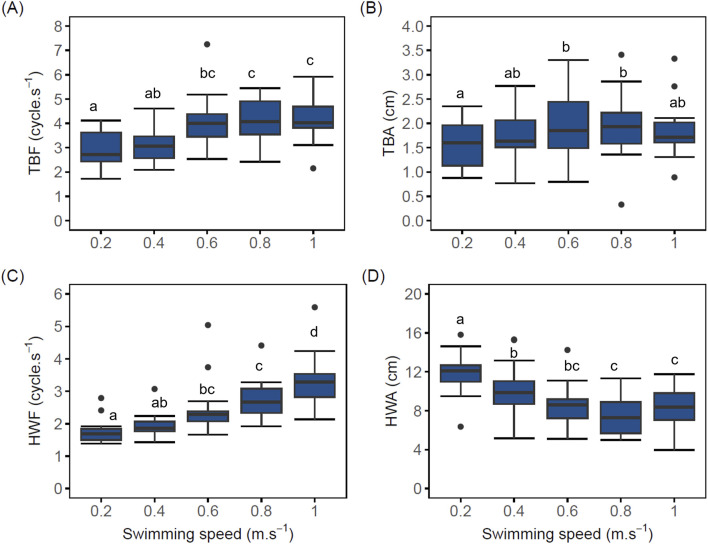
Boxplot of swimming behavior parameters of implanted post-smolt Atlantic salmon (*Salmo salar*; N = 20) swimming at increasing swimming speed. **(A)** Tail beat frequency (TBF) vs. swimming speed (TBF values at speeds of 0.6, 0.8, and 1.0 m.s^–1^ were significantly different compared to the TBF value at 0.2 m.s^–1^ (LMM p < 0.05); **(B)** Tail beat amplitude (TBA) vs. swimming speed (TBA values at speeds of 0.6, 0.8 m.s^–1^, but not 1.0 m.s^–1^, were only significant when compared with 0.2 m.s^–1^ (LMM p < 0.05); **(C)** Head width frequency (HWF) vs. swimming speed (HWF values at speeds of 0.6, 0.8 and 1.0 m.s^–1^ were statistically different when compared to 0.2 m.s^–1^ (LMM p < 0.05), **(D)** Head width amplitude (HWA) vs. swimming speed (HWA values decreased significantly with increasing speeds (LMM p < 0.05). The black dots in the boxplot represent outliers. Different lowercase letters indicate significant difference between values (LMM p < 0.05); same letters denote otherwise. A similar graph for control fish is included in Supplementary Figure S2.

Despite the significant difference in BW between implanted and control fish, neither BW as covariate nor logger implantation as fixed effect had a significant effect on locomotory behavior (Figure 3; Supplementary Figure S2, LMM p > 0.05).

Tail beat frequency (TBF) of implanted fish increased linearly (R^2^ = 0.89) with swimming speed from 2.89 ± 0.17 when swimming at 0.2 m.s^–1^ to 4.18 ± 0.32 cycle.s^–1^ at 1.0 m.s^–1^. From 0.6 to 1.0 m.s^–1^, TBF values of implanted fish (Figure 3A) remained statistically similar (LMM p > 0.05, AIC = 279, Supplementary Table S1). TBF values at swimming speeds of 0.6, 0.8, and 1.0 m.s^–1^ were significantly different compared to the TBF value at 0.2 m.s^–1^ (LMM p < 0.05). Only from 0.4 m.s^–1^ onward did mean TBF correlates with MO_2_ (linear fit R^2^ = 0.87) and its effect on MO_2_ was significant, with an estimated increase of 18.39 mg.kg^–1^.h^–1^ of MO_2_ for each cycle. s^–1^ increase in TBF (LMM 95% CI [5.31, 31.47], t (81) = 2.80, df = 82, p = 0.006).

Although mean tail beat amplitude (TBA) of implanted fish increased with swimming speed from 0.2 up to 0.8 m.s^–1^ (1.57 ± 0.12 to 1.94 ± 0.15 cm), high variations were observed across all swimming speeds (Figure 3B). At 1.0 m.s^–1^, mean TBA dropped to 1.89 ± 0.20 cm. Notwithstanding these high variations, power function provided the most accurate description of the relationship between mean TBA values and swimming speed from 0.2 to 0.8 m.s^–1^ (power fit R^2^ = 0.90, y = 1.95x^0.129^). Effect of swimming speeds of 0.6, 0.8 m.s^–1^, but not 1.0 m.s^–1^, on mean TBA values were only significant when compared with 0.2 m.s^–1^ (LMM p < 0.05, AIC = 138, Supplementary Table S1). The effect of TBA on MO_2_ was statistically non-significant and positive [LMM, beta = 29.47, 95% CI (−1.06, 60.01), t (70) = 1.93, df = 64, p = 0.058].

Head width frequency (HWF) increased linearly with swimming speed from 0.2 to 1.0 m.s^–1^ in implanted fish (linear fit R^2^ = 0.92; Figure 3C). Expected to also reflect respiration, mean HWF of implanted fish correlates linearly and positively with MO_2_ (linear fit R^2^ = 0.76) and this relationship was found to be significant [beta = 38.85, 95% CI (23.86, 53.83), t (81) = 5.16, df = 70, p < 0.001]. In comparison with swimming at 0.2 m.s^–1^, HWF of implanted fish at 0.4 m.s^–1^ was not significant (LMM p > 0.05). However, HWF values at swimming speeds of 0.6, 0.8, and 1.0 m.s^–1^ were statistically different when compared to 0.2 m.s^–1^ (LMM p < 0.05, AIC = 251, Supplementary Table S1).

Mean head width amplitude (HWA) of implanted fish decreased significantly (LMM p < 0.05) and linearly (linear fit R^2^ = −0.74) with increasing speed from 11.94 ± 0.50 cm when swimming at 0.2 m.s^–1^ to 8.38 ± 0.71 cm when swimming at 1.0 m.s^–1^ (Figure 3D). As mean HWA of implanted fish decreased with increasing swimming speed, it stabilized from 0.6 to 1.0 m.s^–1^ with no statistical differences between these values (LMM p > 0.05, AIC = 421, Supplementary Table S1).

### 3.4 Heart rate and acceleration during respirometry

**FIGURE 4:**
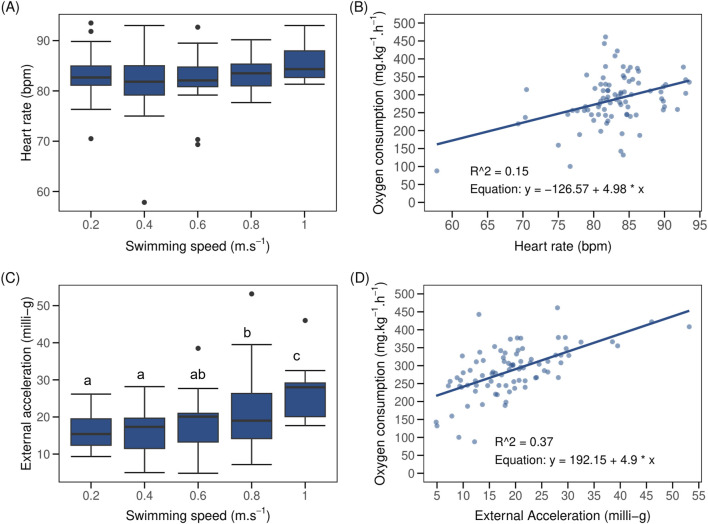
Boxplot (N = 19) of heart rate (HR) and external acceleration (AC) of implant post-smolt Atlantic salmon (*Salmo salar*) vs. swimming speed and linear regression between oxygen consumption (MO_2_) vs. HR and AC of fish in a steady flow swim tunnel. The black dots in the boxplot represent outliers. Shown are **(A)** HR vs. swimming speed which remained more or less stable during the swim-fitness test; **(B)** MO_2_ vs. HR (R^2^ and equation displayed in the figure); **(C)** AC vs. swimming speed (AC values remained statistically similar at 0.2, 0.4 and 0.6 m.s^–1^ but increased significant at 0.8 and 1.0 m.s^–1^; and **(D)** MO_2_ vs. AC (R^2^ and equation displayed in the figure). Different lowercase letters, when present, indicate significant difference between values (LMM p < 0.05); same letters denote otherwise.

When swimming from 0.2 to 1.0 m.s^–1^, HR of implanted fish (N = 19) remained stable between 82.1 ± 1.5 to 84.4 ± 1.1 bpm (Figure 4A). Swimming speed had no significant effect on HR of implanted fish (LMM p > 0.05; marginal R^2^ = 0.02, AIC = 539, Supplementary Table S1). The inclusion of BW, SL, and TL as covariates in the LMM account for 23% of the variations observed in HR of implanted fish (marginal R^2^ = 0.25), even though their influence on HR did not reach statistical significance (LMM p > 0.05, AIC = 535, Supplementary Table S1). Mean HR of implanted fish correlates poorly and positively with MO_2_ (Figure 4B). A single point with low HR and MO_2_ value added far more weight to this correlation than the others that were in a cloud of points without any correlation (R^2^ = 0.06).

Mean AC of implanted fish increased linearly (linear fit R^2^ = 0.90, y = 13.54x + 11.70) from 15.9 ± 1.2 milli-g when swimming at 0.2 m.s^–1^ to 26.7 ± 2.5 milli-g when swimming at 1.0 m.s^–1^ (Figure 4C). This correlated positively with MO_2_ (Figure 4D). Swimming speeds of 0.4 and 0.6 m.s^–1^ had no significant effect (LMM p > 0.05) on AC of implanted fish in comparison to 0.2 m.s^–1^. However, AC of implanted fish increased significantly when swimming at 0.8 and 1.0 m.s^–1^ (LMM p < 0.05, AIC = 573). Inclusion of BW and SL and TL as covariates explained 16% of variation found in the AC of implanted fish, with SL alone accounting for 9% of these variations (marginal R^2^ = 0.37; conditional R^2^ = 0.64, AIC = 569). SL had significant and positive effect on AC (LMM p = 0.02) while the effect of both BW (LMM p = 0.20) and TL (LMM p = 0.08, Supplementary Table S1) were non-significant and negative.

### 3.5 Heart rate during stress challenge tests

**FIGURE 5:**
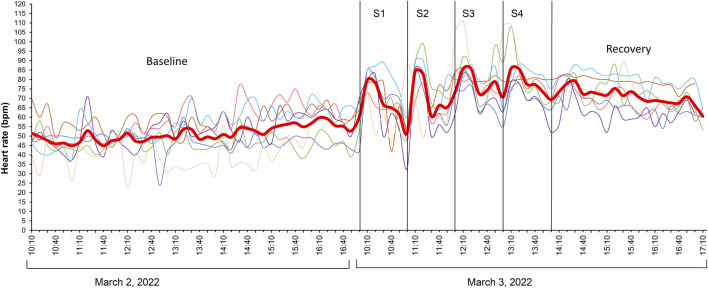
Heart rate (HR) values during the stress challenge test of post-smolt Atlantic salmon (*Salmo salar*; N = 7) implanted with Star-Oddi DST milli-HRT ACT logger. The red line shows the mean HR values corresponding to the Y-axis. Shown on the X-axis is the time period from 10 to 17 h on the day of the stress challenge test and the day before. S1, S2, S3, and S4 represent the four different stressors with ascending level of intensity. HR peaks are statistically higher than baseline values (LMM p < 0.05) ∼20 min after initiating each stress induction but not significantly between each stressor (LMM p > 0.05). A similar graph for acceleration values of fish is included in Supplementary Figure S3.

Baseline HR values of implanted fish (N = 7) in the holding tank 8 days after swim-fitness test and a day prior to stress challenge test was 51 ± 6 bpm (Figure 5). When initiating the first stressor, the HR of fish increased significantly 20 min after initiation to 79 ± 1 bpm (LMM p < 0.05). Subsequently, it decreased to 61 ± 2 bpm during the remaining 40 min. Similarly, significant increases (LMM p < 0.05) and decreases in HR were observed during the second to fourth stages of the stress challenge test, ranging from 84 ± 2 to 86 ± 3 bpm and 66 ± 2 to 74 ± 2 bpm respectively when compared to baseline values. Despite the increasing intensity of the four consecutive stressors, HR values remained statistically similar between stressors (LMM p > 0.05). Three hours after the stress challenge test, HR of fish remained high and showed only slow decrease from 79 ± 2 to 61 ± 2 bpm.

### 3.6 Cortisol concentrations during the stepwise stress challenge test

As we challenged fish (N = 7) to the four sequential stressors, cortisol levels in the water sample increased from a baseline level of 133 pg.L^–1^; 212 pg.L^–1^ at step 1; 388 pg.L^–1^ at step 2; 345 pg.L^–1^ at step 3, and 362 pg.L^–1^ at step 4.

## 4 Discussion

This study used DST milli-HRT data loggers to record the HR and AC of post-smolt Atlantic salmon during a swim-fitness and stress challenge test. These parameters were recorded simultaneously with determinations of oxygen consumption and locomotory behavior parameters. We compared swimming performance variables of implanted fish to values of the control group without implanted loggers to ensure that there was no significant impact of logger implantation on the swimming capacity of fish. We tested two hypotheses on Atlantic salmon (∼471 g) swimming in steady flow: condition (i) With increasing swimming speed, the HR and AC of Atlantic salmon will increase alongside MO_2_, as the HR should ensure sufficient oxygen delivery to the red muscles, and (ii) In response to induced stress, Atlantic salmon will show increased HR and AC, reflecting their physiological adaptations to stress coping.

First, we showed that implanted fish had similar U_crit_ values as their controls (0.84 vs. 0.83 m.s^–1^), as well as similar U_opt_ values (0.72 vs. 0.71 m.s^–1^) and minimum COT values (87 vs. 91 mg.kg^–1^.km^–1^), respectively (Figure 1). Our U_crit_ values were comparable to those reported by Hvas et al. (2021a), who used a similar swimming protocol and HR–AC loggers. For their study, the 950 g Atlantic salmon swam in groups, unlike the individually swimming fish in our study. Hvas et al. (2021a) reported on U_crit_ values of 1.11 and 1.15 m.s^–1^ for implanted and control fish, respectively. Our results showed statistically similar MO_2_ (Figure 2; Supplementary Figure S1) and locomotory parameters measured (Figure 3; Supplementary Figure S2) between implanted and control fish. Under resting at minimal flow conditions and when swimming at a speed of 0.2 m.s^–1^, we found higher MO_2_ values as compared to swimming at 0.4 m.s^–1^ in both implanted and control fish (Figure 2; Supplementary Figure S1). This is likely caused by introducing the fish in the swim tunnels and housing them individually leading to an elevated stress level, resulting in a hypoxic ventilatory response where fish increase their ventilatory activities to improve gill diffusion conductance (Borch et al., 1993; Abdel-Tawwab et al., 2019). The range of MO_2_ values obtained in this study aligns with those found in other studies (Eliason et al., 2013; Hvas et al., 2021b; Hvas and Oppedal, 2017) and our previous research (Agbeti et al., 2024), where Atlantic salmon (∼275 g) swam in a 30 L Loligo swim tunnel at speeds ranging from 0.2 to 0.8 m.s^–1^ under steady flow conditions. Results support the MO_2_ and swimming speed relationship that we previously observed. Our current findings also show that the data loggers that were implanted into the cavity of Atlantic salmon did not place a significant burden on the fish and were therefore suitable for use in this physiological research.

In line with the MO_2_ values, also HR was already high when fish were introduced in the tunnels which may be the result of anesthesia, handling and, particularly for this species, individual housing. These initial high HR may have masked any correlation between MO_2_ and HR at low swimming speeds. Values when swimming at 0.2 m.s^–1^ were 82.1 ± 1.5 bpm and significantly higher than the baseline values of 51 ± 6 bpm as measured when swimming freely and group-wise in the tanks. Similarly, Sandrelli and Gamperl (Sandrelli and Gamperl, 2023) reported a lower resting HR for ∼840 g Atlantic salmon swimming freely in holding tank (∼49 bpm) than those in swim tunnel (∼69 bpm). HR of implanted fish remained stable across the range of swimming speeds tested (Figure 4A). Despite the increased swimming effort, reflected by significant changes in MO_2_ (Figure 2), AC (Figure 4C), and tail and head width frequencies (Figures 3A, C), the change in HR was minimal, approximately 2.3 bpm, corresponding to a factorial HR scope of 1.03. Hvas et al. (2021a) observed a factorial HR scope of 1.3 in Atlantic salmon (∼1000 g) when swimming speed increased from 0.3 to 1.1 m.s^–1^, and a scope of 2.1 when using nighttime resting heart rate as a baseline. In our study, a factorial scope of 1.7 was observed when comparing maximum HR with baseline HR in holding tanks. A similar HR scope of 1.26 was found in Atlantic salmon (∼740 g) swimming from 0.3 to 0.75 m.s^–1^ (Zrini and Gamperl, 2021). Adult sockeye salmon, *Oncorhynchus nerka*, (∼2.5 kg) showed a factorial HR scope of 1.02–1.39 during a repeated U_crit_ test (Eliason et al., 2013). For yellowtail kingfish, a factorial HR scope of 1.3 was extrapolated based on HR values reported when the fish swam at speeds ranging from 0.4 to 1.0 m.s^–1^ (Palstra et al., 2024). Some studies have found strong positive relationships between HR and MO_2_ in fish species such as pike (*Esox lucius*) [R^2^ = 0.89 (Armstrong, 1986)], sockeye salmon [R^2^ = 0.70 (Clark et al., 2010)], but also in Atlantic salmon [R^2^ = 0.82–0.88 (Lucas, 1994)], However, in our study, HR explained only about 15% of the variation in MO_2_ (Figure 4B). Our findings are consistent with the suggestion that salmonids may not solely rely on HR to increase cardiac output to supply oxygen to the red muscles (Hvas et al., 2021a; Thorarensen et al., 1996; Yousaf et al., 2024; Bloecher et al., 2024). Salmonids can increase blood flow by increasing the stroke volume, which is the volume of blood pumped out by the heart with each contraction. This reduces the need to change HR to regulate blood flow to tissues, especially during strenuous activity (Thorarensen et al., 1996). Thus, increases in oxygen consumption and cardiac output in salmonids may be more influenced by changes in stroke volume and perhaps oxygen uptake efficiency by the tissues than by changes in HR (Eliason et al., 2013; Thorarensen et al., 1996; Kolok and Farrell, 1994). Eliason et al. (2013) found that during repeated U_crit_ tests, the stroke volume scope of 2.4 and cardiac output scope of 2.8 were twice as high as the HR scope of 1.0–1.4. In other marine fish species like the yellowfin tuna (*Thunnus albacares*), known for its athletic swimming abilities, an increase in HR and a decrease in stroke volume was reported to increase cardiac output at swimming speeds of 1.2–2.1 FL.s^–1^ (Korsmeyer et al., 1997). This range illustrates the intra-species variability in how HR and stroke volume contribute to cardiac output. In our study, we further exposed Atlantic salmon in a holding tank to four consecutive stressors, each more intense than the previous one, and observed significantly higher HR peaks compared to baseline values (Figure 5). Mean HR values of fish were similar regardless of the stress intensity. This increase in HR during each stress induction illustrates an immediate physiological response of fish heart to stress stimuli. This could be primarily driven by the release of stress hormones such as catecholamines and cortisol which prepare fish to either engage in fight or flight response by increasing oxygen and nutrient delivery to vital organs (Sadoul and Geffroy, 2019). Indeed, we found that the peaks in HR, 20 min after the initiation of each of the four stressors, corresponded to high water cortisol levels as compared to baseline value. The rapid decrease in HR during recovery, to similar values as baseline values after the first stressor, signifies the fish’s return to a state of homeostasis. As the stress load intensified, the HR of fish did not drop to comparable values as initially observed during the first recovery period but rather remained substantially high during the third and fourth recovery periods (Figure 5). Also, after the four induction steps, fish still had not recovered their baseline HR level during 3 h of rest. This suggests that salmon does not recover rapidly when subjected to repeated stress, and it is probable that plasma cortisol levels remained high during recovery periods between each stressor. For comparison, previously, we have performed a similar stress challenge to yellowtail kingfish (652 ± 152 g; 24°C) that were implanted with the same loggers (Palstra et al., 2024). We observed that HR responses during the first, second and third stressors remained similar, ranging from 138 to 144 bpm. However, HR increased substantially during the fourth stressor, reaching 186 bpm. These HR values were nearly twice as high as the values recorded in this study using post-smolt Atlantic salmon.

Future research should include additional measurements, such as stroke volume, alongside HR, to better understand their relationship. Such investigations could offer a more comprehensive understanding of how increased oxygen demand is met in Atlantic salmon and the roles of HR and stroke volume during swimming and stress events.

AC of Atlantic salmon, in response to the four stress induction steps, also showed a similar pattern of peaks as observed with HR (Supplementary Figure S3). The first and fourth stressor elicited a freeze response in our fish during recovery, with lower AC values as compared to baseline values. The first stressor, where water level was reduced and immediately filled up, might have triggered a fright response in fish and created an awareness of what is to come. Hence, during the second recovery period, AC values were not lower but comparable to baseline values. The fourth stressor, where fish were chased for 5 min before water was restored to its original level, may have completely fatigued the fish, resulting further in lower AC values during recovery (Svendsen et al., 2021) when compared to baseline AC values. Despite the intensity of the fourth stressor and the passive behavior observed afterwards, AC values, upon recovery, returned to comparable levels with baseline after 3 h of rest. During the swim fitness test, we found that AC of Atlantic salmon increased by 1.9-fold when swimming from 0.2 to 1.0 m.s^–1^ with some individual fish reaching maximum AC values of 53 milli-g (Figure 4C). This is comparable to the reported factorial change of 2.5 by Zrini and Gamperl (Zrini and Gamperl, 2021) in ∼740 g Atlantic salmon subjected to a similar exercise test ranging from 0.3 to 1.0 m.s^–1^. Other athletic finfish species, such as European sea bass (*Dicentrarchus labrax*), can increase their AC by a factor of 6 when swimming at increasing speeds (Wright et al., 2014). In Sockeye salmon swimming in a respirometer, Clark et al. (2010) demonstrated a positive linear relationship between AC and MO_2_ (R^2^ = 0.78). The AC of Atlantic salmon in our study also correlated with MO_2_, explaining close to 40% of the observed variation in oxygen usage as swimming intensity increased (Figure 4D). This suggests an opportunity of using AC data to reliably monitor and estimate metabolic rate of free-swimming fish. We also found a positive correlation between SL and AC, indicating the important role of a muscled tail region in locomotion. It is plausible that the surface area of the caudal peduncle region can strongly correlate with AC and MO_2_. Salmonids, classified as subcarangiform swimmers, swim by bending the posterior section of their bodies and caudal region (Webb, 1984). This aids in generating thrust to overcome drag force which increases with the square of speed (Webb, 1975; Videler, 1993). As far back as in the 1950s, TBF of fishes has been reported to correlate strongly with swimming speed (Bainbridge, 1958). In our study, we found that mean TBF and TBA of implanted fish both increased with increasing swimming speed until the point of exhaustion where mean TBF stabilized (Figure 3A) and mean TBA decreased (Figure 3B). Increases in TBF are in line with AC and MO_2_ (Figures 2, 4C). The linear increase of TBF with swimming speed was also found in our previous research (Agbeti et al., 2024) and in other studies on salmonids (Hvas et al., 2021b; Warren-Myers et al., 2023; Webb et al., 1984). The linear increase of TBF in ∼1000 g salmon, implanted with AC tag, was evident when swimming from 0.3 to 0.8 m.s^–1^ (Warren-Myers et al., 2023). TBA of the fish in our study showed high variability across the whole range of swimming speeds tested and mean TBA increased with swimming speed as a power function. Fish may increase both the amplitude and frequency of their tail movements to generate the thrust needed to overcome drag force. The observed variability in TBA could suggest change in individual fish swimming patterns to minimize speed-specific cost of transport (Li et al., 2021).

In our study, we also measured kinematic variables HWF and HWA. Mean HWF of implanted and control fish increased significantly with increasing swimming speed (but not in phase with the body movements; Figure 3C; Supplementary Figure S2C). This could be indicative of higher respiration rate of the gills to extract sufficient oxygen to cope with the aerobic demands as swimming speed intensifies. Indeed, from the linear mixed model, HWF turned out as a significant predictor of MO_2_. A similar linear increase in HWF with swimming speed was found in yellowtail kingfish, a carangiform swimmer, but it was not significantly correlated with MO_2_ (Palstra et al., 2024). HWA decreased significantly with increasing swimming speed from 0.2 to 0.8 m.s^–1^, suggesting that fish ram-ventilated mostly at higher swimming speeds of 0.6 m.s^–1^ and upwards (Figure 3D; Supplementary Figure S2D). This is done by allowing water to flow continuously over their gills through the mouth without active pumping (Randall, 1982). Higher HWA at lower speed (Figure 3D) may be due to fish bending its body around the dense medium; a phenomenon that became less pronounced as the swimming speed increased. The observed linear decrease in HWA contradicts our previous findings (Agbeti et al., 2024), where an increase in HWA was noted in post-smolt Atlantic salmon with increasing swimming speed when tested in a Loligo swim tunnel. Notably, the swim sectional volume of the Loligo swim tunnel was approximately 14 times smaller than the one used in the current study. It is therefore likely that the modulation of HWA may be influenced by the swim volume in which Atlantic salmon are made to swim, possibly to enhance swimming efficiency. Further investigation is needed to explain this discrepancy.

## 5 Conclusion

HR of post-smolt Atlantic salmon remained high and stable across the range of swimming speeds tested. Despite significant physiological and behavioral responses, such as changes in MO_2_, AC, and tail and head width frequencies, the HR of post-smolt Atlantic salmon showed minimal variation. AC showed a strong correlation with MO_2_ during swim-fitness tests. The increase in MO_2_ also correlated positively and strongly with tail beat and head width frequencies, of which the latter can serve as a proxy for gill respiration. Head width and tail beat frequencies also contributed substantially to the observed variation in MO_2_ but not HR. HR and AC effectively reflected stress induction steps, as evidenced by distinct peaks and may be instrumental to better understand fish behavior under stressful conditions in tank-based fish culture and possibly in offshore aquaculture.

## Data Availability

The original contributions presented in the study are included in the article/Supplementary Material, further inquiries can be directed to the corresponding author.
