# Supplementary material for: Atlantic salmon (Salmo salar) under challenge: Heart rate and acceleration dynamics during exercise and stress
Source: Front Physiol. 2025 Apr 7;16:1562665. doi: 10.3389/fphys.2025.1562665 (PMC12009768; doi:10.3389/fphys.2025.1562665)
Supplement: Supplementary file 1 [file Presentation1.pdf]

Supplementary Material

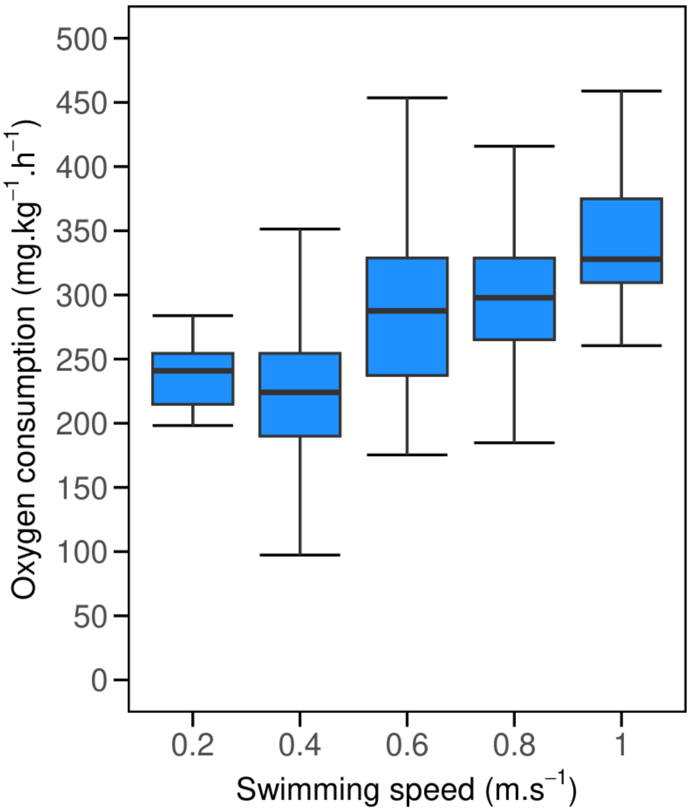

Supplementary Figure S1. Boxplot of oxygen consumption (MO_2) for control post-smolt Atlantic salmon (*Salmo salar*) swimming at increasing swimming speed in a steady flow swim tunnel. Each boxplot represents N = 16 fish. MO_2 increased linearly from 0.4 to 1.0 m.s^{-1}, but plateaued at 0.6 and 0.8 m.s^{-1} before significantly increasing again at 1.0 m.s^{-1} (LMM $p < 0.05$).

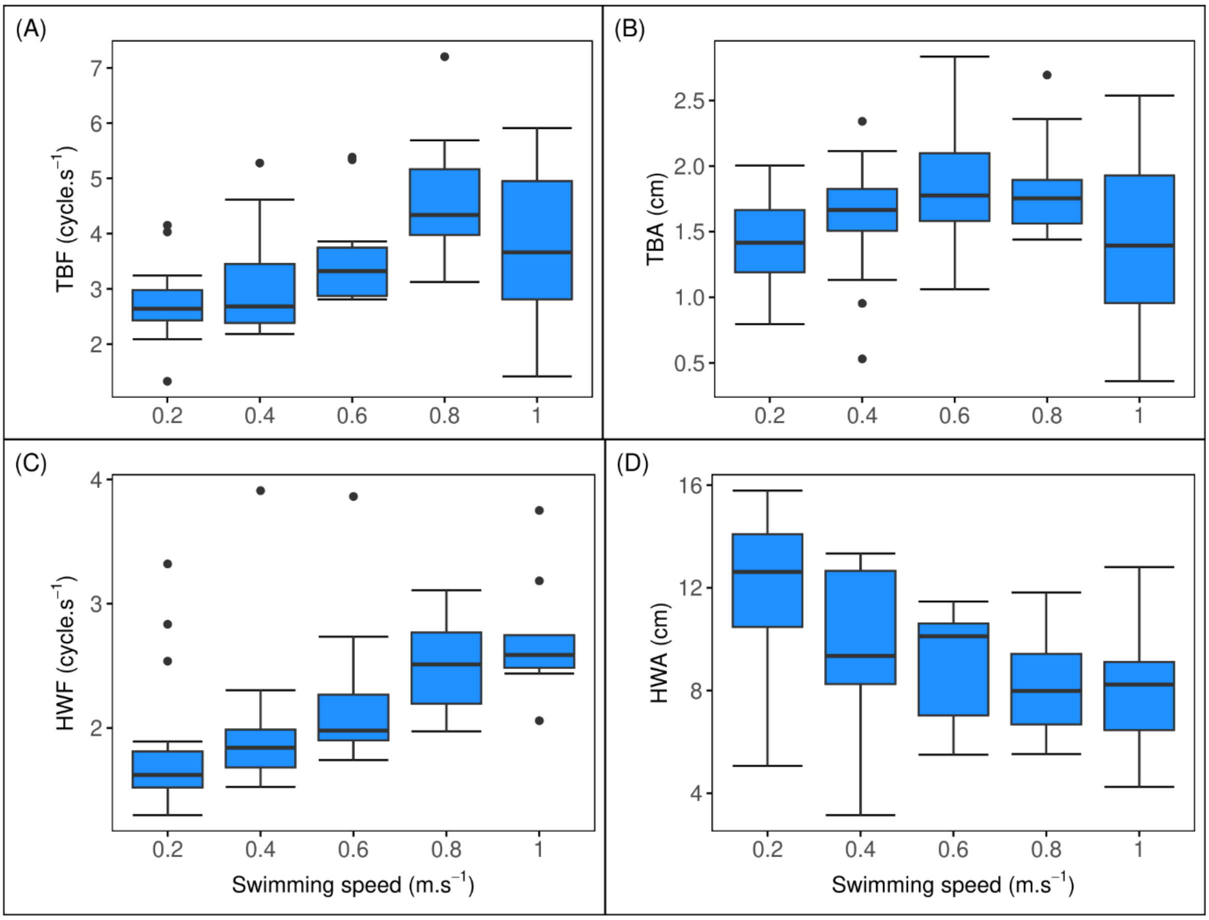

Supplementary Figure S2. Boxplot of swimming behavior parameters of control post-smolt Atlantic salmon (*Salmo salar*; N = 16) swimming at increasing swimming speed. **(A)** Tail beat frequency (TBF) vs. swimming speed; **(B)** Tail beat amplitude (TBA) vs. swimming speed, **(C)** Head width frequency (HWF) vs. swimming speed, **(D)** Head width amplitude (HWA) vs. swimming speed.

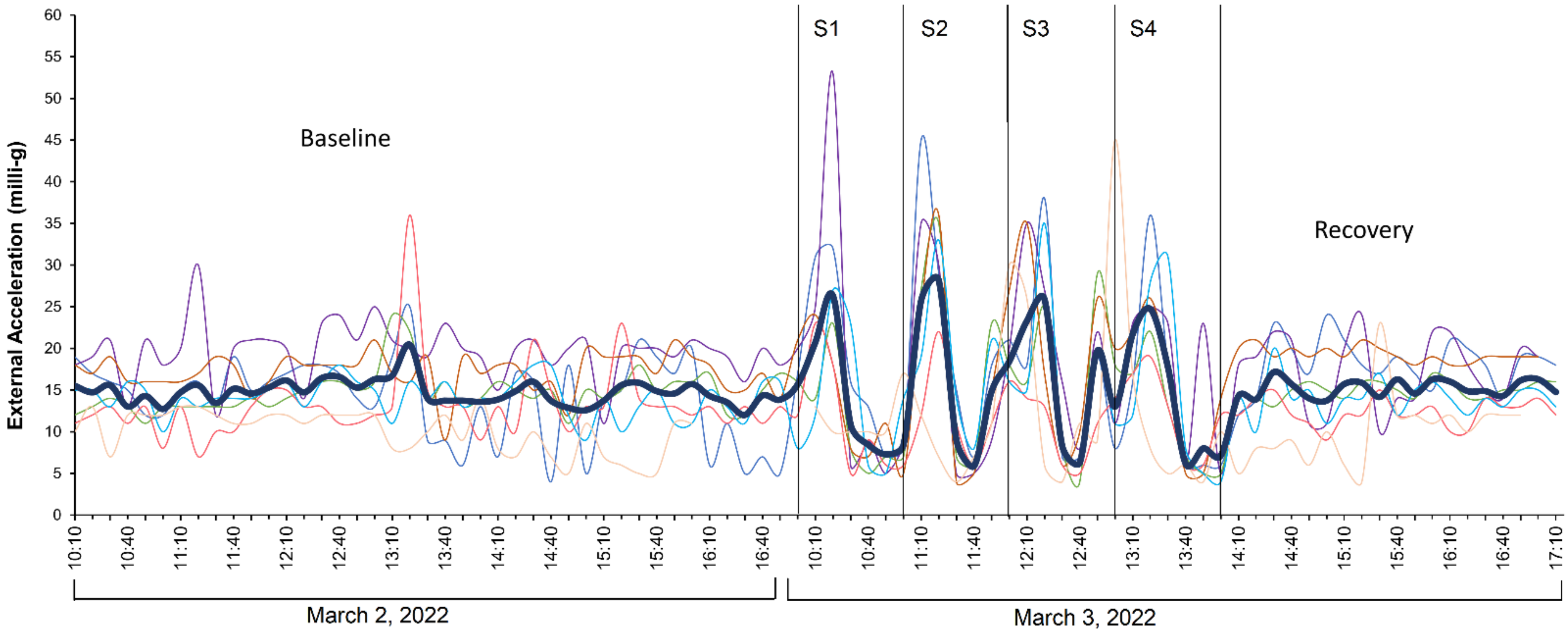

Supplementary Figure S3. External Acceleration (AC) values during the stress challenge test of post-smolt Atlantic salmon (*Salmo salar*; N = 7) implanted with Star-Oddi DST milli-HRT ACT logger. The dark line shows the mean AC values corresponding to the Y-axis. Shown on the X-axis is the time period from 10 to 17 h on the day of the stress challenge test and the day before. S1, S2, S3 and S4 represent the four different stressors with ascending level of intensity. Acceleration peaks are statistically higher than baseline values (LMM, $p < 0.05$) ~20 min after initiating each stress induction but not significantly between each stressor (LMM, $p > 0.05$).

Supplementary Table S1: Linear mixed model (LMM) results for effect of swimming speed, and/or logger implantation on oxygen consumption (MO2), heart rate (HR), External acceleration (AC), tail beat frequency (TBF), tail beat amplitude (TBA), head width frequency (HWF), and head width amplitude (HWA) of post smolt Atlantic salmon (*Salmo salar*). Individual variability was accounted for as random effect in each model. Intercept, coefficient estimate, 95% confidence intervals (CIs), t-value, and the fit values are provided for each model. Standardised parameters were obtained by fitting the model on a standardised version of the dataset. 95% confidence intervals (CIs) and p-values were computed using a Wald t-distribution approximation.

Model	Parameter	Coefficient	95% CI	t	p-value	Effects	Group	Std. Coef.	Std. Coef. 95% CI	Fit
**MO ~ speed +implant +BW +TL +SL+ (1|fishID)**	(Intercept)	54.03	[-381.53, 489.59]	0.25	0.807	fixed		-0.3	[-0.71, 0.10]	
	speed [0.4]	-25.28	[-49.04, -1.52]	-2.1	0.037	fixed		-0.35	[-0.68, -0.02]	
	speed [0.6]	31.5	[7.66, 55.35]	2.61	0.01	fixed		0.44	[0.11, 0.77]	
	speed [0.8]	37.33	[13.69, 60.97]	3.12	0.002	fixed		0.52	[0.19, 0.85]	
	speed [1]	100.4	[71.16, 129.63]	6.79	< .001	fixed		1.4	[0.99, 1.81]	
	Logger implant	1.84	[-35.09, 38.78]	0.1	0.922	fixed		0.03	[-0.49, 0.54]	
	BW	-0.01	[-0.55, 0.52]	-0.04	0.966	fixed		-0.008	[-0.39, 0.37]	
	TL	-61.24	[-108.63, -13.86]	-2.55	0.012	fixed		-1.22	[-2.17, -0.28]	
	SL	75.25	[16.90, 133.59]	2.55	0.012	fixed		1.33	[0.30, 2.36]	
		37.31				random	fishID			
		49.11				random	Residual			
	AIC									1640.78
	AICc									1642.63
	BIC									1674.18
	R^2 (conditional)									0.57
	R^2 (marginal)									0.32

**MO ~ speed +BW +TL +SL+ (1|fishID)**	(Intercept)	-125.93	[-728.95, 477.08]	-0.42	0.677	fixed		-0.67	[-1.08, -0.27]	
	speed [0.6]	56.34	[22.55, 90.13]	3.34	0.001	fixed		0.74	[0.30, 1.18]	
	speed [0.8]	62.9	[29.68, 96.12]	3.79	< .001	fixed		0.83	[0.39, 1.26]	
	speed [1]	120.51	[80.38, 160.64]	6.01	< .001	fixed		1.58	[1.06, 2.11]	
	BW	0.03	[-0.76, 0.83]	0.08	0.936	fixed		0.02	[-0.49, 0.53]	
	TL	-54.92	[-127.54, 17.70]	-1.51	0.135	fixed		-1.17	[-2.71, 0.38]	
	SL	72.42	[-18.03, 162.86]	1.6	0.114	fixed		1.36	[-0.34, 3.07]	
		51.15				random	Residual			
		43.54				random	fishID			
	AIC									708.39
	AICc									711.54
	BIC									728.23
	R^2 (conditional)									0.61
	R^2 (marginal)									0.33
**TBF~speed + + (1|fishID)**	(Intercept)	2.96	[2.49, 3.43]	12.5	< 0.001	fixed		-0.7	[-1.09, -0.30]	
	speed [0.4]	0.42	[-0.12, 0.97]	1.54	0.128	fixed		0.36	[-0.11, 0.82]	
	speed [0.6]	1.11	[0.56, 1.66]	4.01	< 0.001	fixed		0.94	[0.47, 1.40]	
	speed [0.8]	1.24	[0.69, 1.79]	4.5	< 0.001	fixed		1.05	[0.59, 1.52]	
	speed [1]	1.51	[0.90, 2.12]	4.89	< 0.001	fixed		1.28	[0.76, 1.80]	
		0.6				random	FishID			
		0.87				random	Residual			
	AIC									279.2
	AICc									280.5
	BIC									297

	R^2 (conditional)									0.47
	R^2 (marginal)									0.21
**TBA ~speed + BW + TL + SL + (1|fishID)**	(Intercept)	-2.82	[-7.65, 2.00]	-1.16	0.248	fixed		-0.42	[-0.81, -0.02]	
	speed [0.4]	0.21	[0.00, 0.41]	2.02	0.046	fixed		0.35	[0.01, 0.70]	
	speed [0.6]	0.38	[0.18, 0.59]	3.75	< .001	fixed		0.65	[0.31, 1.00]	
	speed [0.8]	0.4	[0.20, 0.60]	3.9	< .001	fixed		0.68	[0.33, 1.03]	
	speed [1]	0.21	[-0.02, 0.44]	1.8	0.075	fixed		0.35	[-0.04, 0.74]	
	BW	2.71E-03	[0.00, 0.01]	0.82	0.414	fixed		0.23	[-0.32, 0.77]	
	TL	-0.3	[-0.88, 0.28]	-1.03	0.307	fixed		-0.82	[-2.41, 0.77]	
	SL	0.43	[-0.28, 1.15]	1.2	0.232	fixed		1.07	[-0.70, 2.83]	
		0.4				random	FishID			
		0.32				random	Residual			
	AIC									137.76
	AICc									140.41
	BIC									163.19
	R^2 (conditional)									0.72
	R^2 (marginal)									0.3
**HWF~speed + (1|fishID)**	(Intercept)	1.73	[1.36, 2.10]	9.27	< .001	fixed		-0.68	[-1.03, -0.34]	
	speed [0.4]	0.19	[-0.33, 0.72]	0.73	0.466	fixed		0.18	[-0.31, 0.67]	
	speed [0.6]	0.71	[0.18, 1.23]	2.69	0.009	fixed		0.66	[0.17, 1.15]	
	speed [0.8]	1.02	[0.49, 1.54]	3.85	< .001	fixed		0.95	[0.46, 1.43]	
	speed [1]	2.09	[1.52, 2.65]	7.33	< .001	fixed		1.94	[1.42, 2.47]	
		0.06				random	FishID			
		0.83				random	Residual			

	AIC									251.6
	AICc									252.88
	BIC									269.47
	R^2 (conditional)									0.41
	R^2 (marginal)									0.41
**HWA~speed + (1|fishID)**	(Intercept)	11.94	[10.93, 12.95]	23.53	< 0.001	fixed		0.99	[0.62, 1.36]	
	speed [0.4]	-2.02	[-3.22, -0.83]	-3.37	0 0.001	fixed		-0.74	[-1.17, -0.30]	
	speed [0.6]	-3.61	[-4.81, -2.42]	-6.02	< 0.001	fixed		-1.32	[-1.75, -0.88]	
	speed [0.8]	-4.45	[-5.64, -3.25]	-7.4	< 0.001	fixed		-1.62	[-2.06, -1.19]	
	speed [1]	-3.94	[-5.24, -2.63]	-6	< 0.001	fixed		-1.44	[-1.91, -0.96]	
		1.24				random	FishID			
		1.9				random	Residual			
	AIC									420.54
	AICc									421.82
	BIC									438.41
	R^2 (conditional)									0.54
	R^2 (marginal)									0.35
**HR ~speed + (1|fishID)**	(Intercept)	82.29	[79.71, 84.87]	63.52	< .001	fixed		-0.05	[-0.51, 0.42]	
	speed 0 [0.4]	-0.64	[-3.27, 1.99]	-0.48	0.63	fixed		-0.12	[-0.59, 0.36]	
	speed 0 [0.6]	-0.58	[-3.21, 2.06]	-0.44	0.664	fixed		-0.1	[-0.58, 0.37]	

	speed 0 [0.8]	0.89	[-1.75, 3.52]	0.67	0.505	fixed		0.16	[-0.31, 0.63]	
	speed 0 [1]	1.4	[-1.50, 4.30]	0.96	0.341	fixed		0.25	[-0.27, 0.77]	
		3.9				random	fishID			
		4.08				random	Residual			
	AIC									538.74
	AICc									540.1
	BIC									556.24
	R^2 (conditional)									0.49
	R^2 (marginal)									0.02
**HR ~speed + BW + TL + SL + (1|fishID)**	(Intercept)	35.85	[-4.96, 76.65]	1.75	0.084	fixed		-0.04	[-0.45, 0.38]	
	speed 0 [0.4]	-0.64	[-3.27, 1.99]	-0.48	0.629	fixed		-0.12	[-0.59, 0.36]	
	speed 0 [0.6]	-0.58	[-3.21, 2.05]	-0.44	0.663	fixed		-0.1	[-0.58, 0.37]	
	speed 0 [0.8]	0.89	[-1.74, 3.52]	0.67	0.504	fixed		0.16	[-0.31, 0.63]	
	speed 0 [1]	1.37	[-1.53, 4.27]	0.94	0.349	fixed		0.25	[-0.27, 0.77]	
	BW	0.03	[-0.02, 0.09]	1.21	0.228	fixed		0.28	[-0.18, 0.75]	
	TL	-1.11	[-6.00, 3.78]	-0.45	0.653	fixed		-0.32	[-1.71, 1.08]	
	SL	2.22	[-3.88, 8.32]	0.73	0.47	fixed		0.56	[-0.98, 2.11]	
		2.94				random	fishID			
		4.08				random	Residual			
	AIC									534.94
	AICc									537.73
	BIC									559.94
	R^2 (conditional)									0.52
	R^2 (marginal)									0.26

**AC ~speed + BW + TL + SL + (1|fishID)**	(Intercept)	-68.29	[-130.85, -5.74]	-2.17	0.033	fixed		-0.39	[-0.78, 0.00]	
	speed 0 [0.4]	0.4	[-3.16, 3.97]	0.23	0.822	fixed		0.05	[-0.37, 0.47]	
	speed 0 [0.6]	2.46	[-1.10, 6.03]	1.38	0.173	fixed		0.29	[-0.13, 0.71]	
	speed 0 [0.8]	5.93	[2.37, 9.50]	3.31	0.001	fixed		0.7	[0.28, 1.12]	
	speed 0 [1]	12.21	[7.94, 16.48]	5.69	< .001	fixed		1.44	[0.93, 1.94]	
	BW	-0.05	[-0.14, 0.03]	-1.3	0.198	fixed		-0.31	[-0.78, 0.16]	
	TL	-6.82	[-14.35, 0.71]	-1.8	0.075	fixed		-1.29	[-2.71, 0.13]	
	SL	11.07	[1.67, 20.47]	2.35	0.022	fixed		1.85	[0.28, 3.41]	
		4.7				random	fishID			
		5.52				random	Residual			
	AIC									568.71
	AICc									571.61
	BIC									593.37
	R^2 (conditional)									0.64
	R^2 (marginal)									0.37
